# Investigating trehalose synthesis genes after cold acclimation in the Antarctic nematode *Panagrolaimus* sp. DAW1

**DOI:** 10.1242/bio.023341

**Published:** 2017-11-24

**Authors:** Anna C. Seybold, David A. Wharton, Michael A. S. Thorne, Craig J. Marshall

**Affiliations:** 1 Department of Biochemistry, University of Otago, Dunedin 9054, New Zealand; 2 Department of Zoology, University of Otago, Dunedin 9054, New Zealand; 3 British Antarctic Survey, Natural Environment Research Council, Cambridge, CB3 0ET, United Kingdom; 4 Genetics Otago, University of Otago, Dunedin 9054, New Zealand

**Keywords:** *Panagrolaimus davidi*, *Panagrolaimus* sp. DAW1, Trehalose, *Tps-2*, Cryoprotective dehydration, Freezing tolerance

## Abstract

*Panagrolaimus* sp. DAW1 is a freeze-tolerant Antarctic nematode which survives extensive intracellular ice formation. The molecular mechanisms of this extreme adaptation are still poorly understood. We recently showed that desiccation-enhanced RNA interference (RNAi) soaking can be used in conjunction with quantitative polymerase chain reaction (qPCR) to screen for phenotypes associated with reduced expression of candidate genes in *Panagrolaimus* sp. DAW1. Here, we present the use of this approach to investigate the role of trehalose synthesis genes in this remarkable organism. Previous studies have shown that acclimating *Panagrolaimus* sp. DAW1 at 5°C before freezing or desiccation substantially enhances survival. In this study, the expression of *tps-2* and other genes associated with trehalose metabolism, as well as *lea-1*, *hsp-70* and *gpx-1*, in cold-acclimated and non-acclimated nematodes was analyzed using qPCR. *Pd-tps-2* and *Pd-lea-1* were significantly upregulated after cold acclimation, indicating an inducible expression in the cold adaptation of *Panagrolaimus* sp. DAW1. The role of trehalose synthesis genes in *Panagrolaimus* sp. DAW1 was further investigated by RNAi. Compared to the controls, *Pd-tps-2a(RNAi)*-treated and cold-acclimated nematodes showed a significant decrease in mRNA, but no change in trehalose content or freezing survival. The involvement of two other trehalose synthesis genes (*tps-2b* and *gob-1*) was also investigated. These findings provide the first functional genomic investigation of trehalose synthesis genes in the non-model organism *Panagrolaimus* sp. DAW1. The presence of several trehalose synthesis genes with different RNAi sensitivities suggests the existence of multiple backup systems in *Panagrolaimus* sp. DAW1, underlining the importance of this sugar in preparation for freezing.

## Abstract (precis)

**Summary:** Functional genomics was used to investigate trehalose synthesis genes after cold acclimation in *Panagrolaimus* sp. DAW1, an Antarctic nematode with the ability to survive intracellular freezing.

## INTRODUCTION

The Antarctic nematode, *Panagrolaimus* sp. DAW1 (PaDAW1) is the only multicellular organism known to tolerate intracellular freezing on a routine basis (Wharton and Brown, 1991; Wharton and Ferns, 1995). PaDAW1, formerly known as *Panagrolaimus davidi* CB1, is also tolerant of cryoprotective dehydration (Wharton et al., 2003, 2004a, 2017). These characteristics are adaptations to the nematodes' habitat in and around Antarctic penguin colonies (Raymond et al., 2013a; Wharton and Brown, 1989). During the summer, and perhaps somewhat surprisingly, soils in these regions can warm to above 25°C (Raymond et al., 2013a; Sinclair et al., 2006), and nutrient-rich liquid guano provides an ideal medium for the growth of bacteria on which PaDAW1 feed (Raymond et al., 2013a). PaDAW1 reproduces only when the temperature is above ∼6.8°C (Brown et al., 2004), suggesting that breeding is possible for only a limited time in each season when solar irradiation warms the soil. However, for much of the year, these habitats are both cold and dry with winter temperatures as low as −40°C and very low humidity. PaDAW1 might be considered a temperate nematode with the capacity to tolerate long-term freezing and desiccation.

Survival of freezing and of desiccation may not be obviously related, but both involve the removal of water and concentration of solutes, and tolerance of these states may share similar mechanisms (Storey and Storey, 2013; Teets and Denlinger, 2014). Although PaDAW1 survives both intracellular freezing (Wharton and Ferns, 1995) and cryoprotective dehydration (Wharton et al., 2003), little is known of the mechanisms by which this is achieved. It is clear that survival of intracellular freezing is related to the pattern and distribution of ice formation (Raymond and Wharton, 2016), and that in turn is likely to be associated with osmoregulation within the pseudocoelomic fluid (Wharton, 2010) as well as the presence of ice-active proteins (Barrett et al., 2005). Cryoprotective dehydration seems to occur if freezing rates are slow, but extra- and even intracellular freezing can occur if freezing rates are faster. The increase in freezing rate produces a shift from cryoprotective dehydration to extracellular to intracellular freezing, accompanied by a decrease in survival (Wharton and Ferns, 1995; Wharton et al., 2003). However, many questions remain as to exactly what metabolic changes are responsible for freezing and desiccation tolerance.

The accumulation of the disaccharide, trehalose (α-D-glucopyranosyl-(1→1)-α-D-glucopyranoside), is one of the best characterized metabolic changes during acclimation of anhydrobiotic organisms (Argüelles, 2014; Storey and Storey, 2013). Trehalose protects membranes and proteins from desiccation by replacing structural water (Carpenter et al., 1987; Crowe et al., 1984) and by forming cellular glass (Crowe et al., 1998). Trehalose accumulation has been associated with anhydrobiosis in nematodes such as *Aphelenchus avenae* (Madin and Crowe, 1975), *Anguina tritici* and *Ditylenchus dipsaci* (Womersley and Smith, 1981). In some species, such as *A. avenae*, trehalose accumulation seems to be essential, but not sufficient, for anhydrobiosis (Browne et al., 2004; Higa and Womersley, 1993). In others, such as some anhydrobiotic rotifer and tardigrade species, trehalose accumulation is apparently not essential (Hengherr et al., 2008; Lapinski and Tunnacliffe, 2003). In PaDAW1, a period of acclimation at ∼5°C is associated with both the accumulation of trehalose and a significant increase in survival after subsequent exposure to freezing (Wharton et al., 2000), suggesting that trehalose might play a role in freezing tolerance (Feofilova et al., 2014; Storey and Storey, 2013; Tapia and Koshland, 2014).

Recent work looking at genes expressed during acclimation, freezing and cryoprotective dehydration in PaDAW1 (Thorne et al., 2014) identified a number of trehalose synthesis (tps) genes, late embryogenesis abundant (lea) proteins, heat shock proteins (hsp) and genes associated with antioxidant production that showed evidence of specific upregulation in these conditions (and were distinct from general responses to stress). In the PaDAW1 dataset, two trehalose synthesis genes (*tps* and *gob*), six aquaporin genes and three desaturase genes, as well as nine different lea-type genes and 20 hsp-70 like genes, were identified as potential candidates to be involved in cryoprotective dehydration (Thorne et al., 2014). Broadly similar patterns of expression were seen in the Antarctic nematode *Plectus murrayi* during freezing (Adhikari et al., 2009).

This study investigates expression of selected genes – trehalose-6-phosphate synthase 2 (*tps-2*), trehalose-6-phosphate phosphatase (*gob-1*), late embryogenesis abundant 1 (*lea-1*) protein, glutathione peroxidase 1 (*gpx-1*) and heat shock protein 70 (*hsp-70*) – in cold-acclimated and non-acclimated nematodes using quantitative polymerase chain reaction (qPCR). Previous work has shown that, provided nematodes are properly fed (Raymond and Wharton, 2013), acclimation at 5°C for 3–5 days improves freezing survival from ∼40% to ∼85% (Wharton et al., 2000). This implies that lower temperatures induce physiological and biochemical changes that assist in freezing survival and an increase in trehalose content was a marked example of such a change. Cellular trehalose was shown to increase upon cold acclimation and to be correlated with freezing survival (Wharton et al., 2000). The enzymes trehalose-6-phosphate synthase (TPS) and trehalose-6-phosphate phosphatase (GOB in PaDAW1) are directly involved in trehalose synthesis in nematodes and are essential genes (Avonce et al., 2006; Behm, 1997; Farelli et al., 2014; Pellerone et al., 2003), and the enzyme trehalase is involved in trehalose breakdown (Thorne et al., 2014, 2017; Łopieńska-Biernat et al., 2015).

The role of trehalose and trehalose synthesis genes in PaDAW1 during acclimation at 5°C was further investigated. First, *tps-2a* was silenced by RNA interference (RNAi) and the reduction of mRNA was measured by qPCR, and the amount of trehalose itself was assessed by gas chromatography. Second, the involvement of other trehalose synthesis genes (*tps-2b* and *gob-1*) was investigated using qPCR and RNAi. Third, the freezing survival of *tps-2a,b(RNAi)*-treated nematodes was compared to that of non-treated controls.

## RESULTS

### Identifying candidate genes

**Fig. 1.**
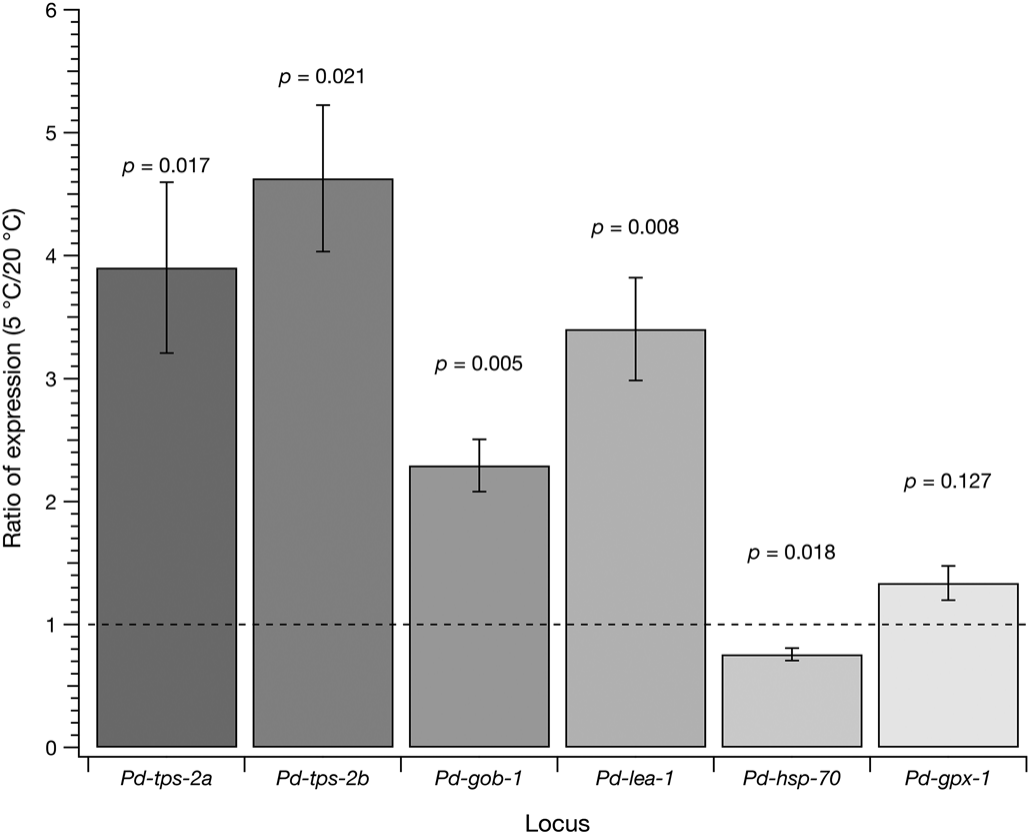
**Analysis of candidate genes in PaDAW1 samples in response to cold acclimation.** (A) Gene expression analysis by qPCR of *Pd-tps-2a*, *Pd-tps-2b*, *Pd-gob-1*, *Pd-lea-1*, *Pd-hsp-70* and *Pd-gpx-1* after acclimation at 5°C for 24 h. Each value represents the mean±s.d. of five biological replicates. Shown is the expression of the 5°C samples relative to the 20°C samples (control), normalized to the value of 1 (dashed line). The statistical significance of differential expression (assessed using *t*-test) is indicated for each gene. (B) Trehalose level in µg/mg (dry weight) measured by gas chromatography in PaDAW1 samples incubated for 4 days at 5°C (acclimated) and 20°C (non-acclimated control). Each value represents the mean±s.d. of four biological replicates.

**Fig. 2.**
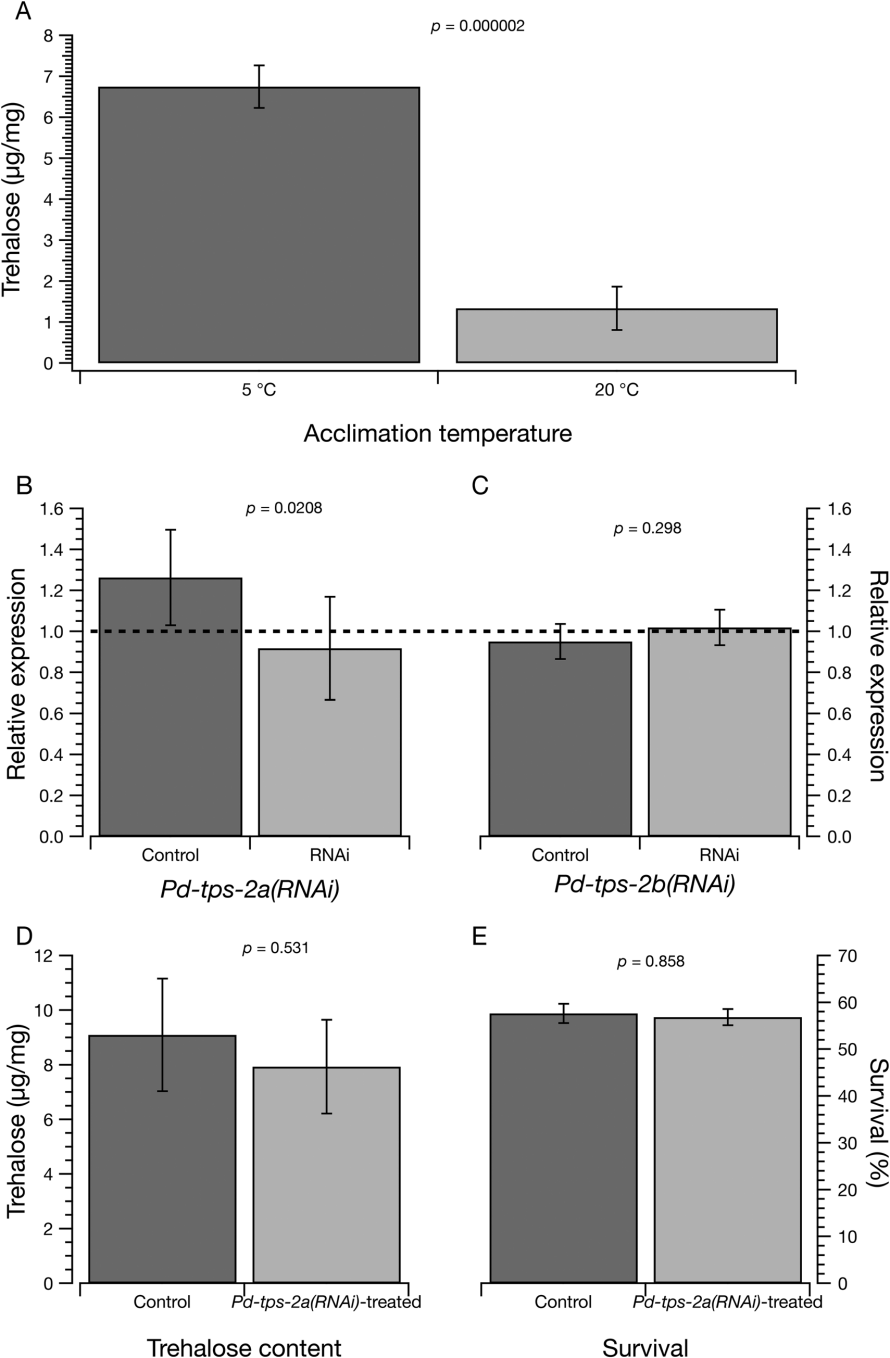
**Analysis of *Pd-tps-2a(RNAi)-*treated and non-treated (control) samples in response to cold acclimation.** (A) Trehalose content of nematodes acclimated at 5°C and 20°C for 72 h was determined by gas chromatography and expressed as µg trehalose/mg of nematode dry weight. (B,C) Gene expression of *Pd-tps-2a* and *Pd-tps-2b* after RNAi. Relative expression of each locus is shown as for Fig. 1. (D) Trehalose content of control and RNAi-treated nematodes. (E) Freezing survival of control and RNAi-treated nematodes after freezing at –15°C for 30 min as described.

In order to directly examine the relative expression of selected genes in response to acclimation at 5°C, quantitative PCR was performed on PaDAW1 samples maintained at the physiologically significant temperatures of 20°C (active reproduction) and 5°C (active acclimation). This analysis showed a significantly increased expression of four of the six genes tested and a significant reduction of expression of one (Fig. 1). The final gene tested, *Pd-gpx-1*, did not vary significantly. Expression of both *Pd-tps-2a* (relative expression=3.9±0.7, *P=*0.017) and *Pd-tps-2b* (relative expression=4.6±0.6, *P=*0.021) was significantly increased consistent with an increase in trehalose content (Fig. 2A). Trehalose-6-phosphate phosphatase (*Pd-gob-1*), an enzyme also involved in trehalose metabolism (relative expression=2.3±0.2, *P*=0.005) and *Pd-lea-1* (relative expression=3.4±0.4, *P=*0.008) were significantly upregulated in the 5°C samples. *Pd-gpx-1* was slightly upregulated in the 5°C samples compared to the 20°C samples but this was not statistically significant (relative expression=1.33±0.14, *P=*0.13). In contrast, *Pd-hsp-70* was slightly and significantly downregulated (relative expression=0.75±0.05, *P=*0.018).

The upregulation of *Pd-tps-2* expression upon cold acclimation in PaDAW1 was correlated with an increase in total trehalose content. Our results showed that the trehalose level was significantly higher in cold-acclimated samples (6.8±0.5 µg/mg at 5°C versus 1.3±0.5 µg/mg at 20°C, a 5.2-fold increase, *P*=2×10^−6^) (Fig. 2A). These data show a large and significant increase in trehalose content in response to acclimation at 5°C, as noted previously (Wharton et al., 2000). This suggests that trehalose may be one of the agents responsible for the correlation between cold acclimation and freezing survival.

### Can RNAi efficiently silence tps-2 expression and is there any phenotype?

Since gene expression analysis by qPCR showed a significant upregulation of both *Pd-tps-2a* and *Pd-tps-2b* in cold-acclimated samples (Fig. 1A), these loci were chosen for further analysis by RNAi. Expression of *Pd-tps-2a* assessed by qPCR after treatment with *Pd-tps-2a(RNAi)* showed a decrease relative to control RNAi-treated nematodes of *Pd-tps-2* [RNAi relative expression of 0.92±0.25, *P=*0.021 versus control relative expression 1.26±0.26, overall relative expression (RNAi/Control)=0.73; Fig. 2B]. It is not clear if this difference is biologically significant even though it is statistically significant at *P*<0.05. Such small differences of expression may not be enough to generate a phenotype (Fraser et al., 2000).

RNAi of *Pd-tps-2b* (RNAi relative expression of 1.02±0.09, *P=*0.021 versus control relative expression 0.95±0.09, overall relative expression=1.07; Fig. 2C) showed no significant decrease in expression. To determine whether a small change in TPS-2 synthesis was associated with any change in trehalose content, we measured the amount of trehalose in nematodes after RNAi treatment. No significant difference in the trehalose content between *Pd-tps-2a(RNAi)*-treated (9.1±2.1 µg/mg) and non-treated control samples (7.9±1.7 µg/mg) was detected (Fig. 2D). We also measured freezing survival and found no difference between RNAi-treated (57.7%±10.3%) and control samples (56.8%±12.1%) (Fig. 2E).

## DISCUSSION

### Investigating candidate genes

**Fig. 3.**
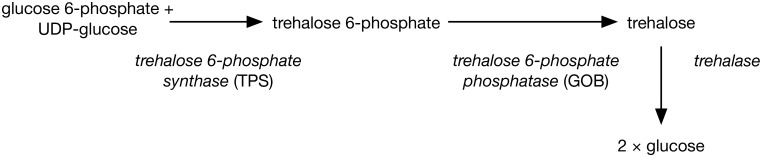
**Schematic outline of trehalose synthesis and degradation in nematodes.** Adapted from Behm (1997).

Candidate genes were selected from those thought to be associated with cold tolerance in nematodes (Adhikari et al., 2009) and using transcriptomic data from PaDAW1 (Thorne et al., 2014, 2017). Three of these are associated with trehalose synthesis and this is consistent with previous findings that cold acclimation of PaDAW1 under these conditions is correlated with a significant increase in trehalose content (Wharton et al., 2000). Synthesis of trehalose in metazoans (which include nematodes) occurs in two steps involving the formation of trehalose 6-phosphate from glucose 6-phosphate and UDP-glucose, followed by the removal of phosphate to form trehalose (Fig. 3). The enzymes involved are TPS and trehalose-6-phosphate phosphatase (GOB), and trehalase in the breakdown of trehalose (Behm, 1997). Our data show a significant upregulation of both *Pd-tps-2a* and *Pd-lea-1*, whereas *Pd-gpx-1* and *Pd-hsp-70* showed no significant change in mRNA levels.

The observation that *Pd-tps-2a* and *Pd-lea-1* are significantly upregulated upon cold acclimation, suggests that these two genes are cold inducible and therefore likely to be involved in cold adaptation of PaDAW1. Late embryogenesis proteins (lea) were originally described in plants (Tunnacliffe et al., 2010) and are now known to have a role in preventing protein aggregation in a wide range of organisms including nematodes (Hand et al., 2016; Yaari et al., 2016). Two other genes, *Pd-gpx-1* and *Pd-hsp-70*, involved in oxidative metabolism and protein folding, respectively, remained nearly unchanged after cold acclimation, indicating that they might be constitutively expressed in PaDAW1. Similar expression patterns in response to desiccation have been demonstrated in other nematode species. For example, after cold acclimation in *P. murrayi*, *Pm-tps* and *Pm-lea* were highly upregulated, while *Pm-gpx* was slightly upregulated and *Pm-hsp-70* remained unchanged (Adhikari et al., 2009). Similarly, in *Panagrolaimus superbus*, *Ps-lea* was highly upregulated, while *Ps-gpx* was slightly upregulated and *Ps-hsp-70* remained unchanged (Tyson et al., 2012).

The gene *hsp-70* seems to be upregulated (inducible) in only some nematode species (Choi et al., 2014) and unchanged (constitutive) in others (Tyson et al., 2012). In *P. murrayi*, *hsp-70* is constitutively expressed, contributing to enhanced stress resistance overall. Generally, *hsp* genes are not constitutively expressed but are expressed in response to stress due to incompatibility with normal metabolism, though there are exceptions, such as in notothenioid fish (Buckley and Somero, 2008; Place and Hofmann, 2005). However, *P. murrayi* may have evolved mechanisms to maintain HSP function during normal metabolism in order to survive in an unpredictable environment like Antarctica, with sudden exposure to a variety of stressors (Adhikari et al., 2009). Survival of PaDAW1 may also depend on maintaining constitutive expression of this molecular chaperone.

Oxidative stress is experienced by organisms undergoing a wide range of abiotic stressors, resulting in generation of reactive oxygen species (Reardon et al., 2010). An oxidative stress response has been defined as part of the environmental stress response (Gasch et al., 2000), as well as part of the minimal stress response (Kültz, 2005). In PaDAW1, the antioxidant *gpx-1* was slightly but not significantly upregulated, indicating a minor role or a constitutive expression. The locus, *gpx-1*, has been shown to be upregulated in other nematode species in response to desiccation (Adhikari et al., 2009; Reardon et al., 2010; Tyson et al., 2012). In *Aphelenchus avenae*, *gpx-1* expression was 32 times greater in response to desiccation and RNAi of *gpx-1* reduced desiccation survival in *P. superbus* (Reardon et al., 2010).

In PaDAW1, trehalose accumulation has been shown to correlate with an increase in survival after exposure to freezing. Trehalose may thus play a role in the freeze tolerance of PaDAW1 by protecting membranes against the harmful effects of freeze-induced dehydration (Wharton et al., 2000). Our results showed that the amount of trehalose was significantly higher in cold-acclimated samples than in non-acclimated nematodes. This is consistent with cold acclimation enhancing freezing survival in PaDAW1 (Raymond et al., 2013; Wharton et al., 2000).

### RNAi affects tps-2 expression but not trehalose synthesis

Gene expression analysis by qPCR showed a significant upregulation of *Pd-tps-2* in cold-acclimated samples compared to non-acclimated samples. Furthermore, the level of trehalose, the product of TPS-2 activity, is increased 4.4-fold in response to cold acclimation. Therefore, *Pd-tps-2* was chosen for further analysis by RNAi. Gene expression analysis by qPCR showed a slight but significant downregulation of *Pd-tps-2a(RNAi)-*treated samples compared to the non-treated samples.

However, no significant difference in trehalose content between *Pd-tps-2a(RNAi)*-treated and non-treated samples was found. The absence of any change in trehalose content could be caused by a number of things. One possibility is that the pool of trehalose is large enough that changes in synthesis over 72 h are not detectable. This seems unlikely since acclimation at 5°C for 96 h increases trehalose content by ∼4.4-fold (Fig. 1B). Alternatively, *tps-2* may not be efficiently silenced. This could be a reflection of a limited reduction in mRNA content [only ∼25% in our data (Fig. 2B)] or because there are other genes for trehalose synthesis. According to Pellerone et al. (2003), less than 100% knockdown of *tps* expression could provide enough enzyme activity to allow residual trehalose metabolism. Therefore, a significant reduction in mRNA may not alter the amount of the enzyme enough to detect a phenotype even though RNAi might have reduced the amount of the target mRNA.

There is evidence that PaDAW1 has more than one *tps* gene which could compensate for the loss of expression at one locus. In *Caenorhabditis* *elegans*, a reduction in trehalose level of >90% (confirmed by qPCR) was achieved after a double knockdown of both *tps* genes. However, no loss-of-function phenotypes were observed (Pellerone et al., 2003). It is therefore questionable whether a phenotype, such as a decrease in survival after exposure to freezing, would be observable in PaDAW1, particularly since the trehalose level was not decreased after *Pd-tps-2a* knockdown. It is also possible, that the half-life of trehalose is significantly longer than the RNAi effect, complicating detection of a decrease in trehalose levels.

### PaDAW1 has multiple genes for trehalose synthesis

In contrast to *Pd-tps-2a*, gene expression analysis of *Pd-tps-2b(RNAi)*-treated samples showed no significant downregulation of the target mRNA compared to the non-treated samples. The fact that *Pd-tps-2a*, but not *Pd-tps-2b*, is sensitive to RNAi is interesting and indicates that these genes probably act differently. They have either evolved different features or are expressed in different tissues with different accessibility to environmental RNAi. Expression in different tissues has been described for trehalase genes, where membrane associated and soluble *tre* activities have been observed (reviewed in Behm, 1997).

Conant and Wagner (2004) found that mutational robustness is greatest for closely related gene duplicates. Since duplicate genes often have similar functions, the loss of one duplicate can be tolerated because other copies can buffer against this loss. They also found a positive correlation between the amino acid distance and the number of duplicates with different knockdown effects (Conant and Wagner, 2004). Thus, the more distant two duplicates are, the more likely it is that one has a stronger knockdown effect than the other. This observation might explain our data on inhibition of *tps-2a* and *tps-2b* synthesis. Symmetric divergence, which probably increases with amino acid distance and divergence time, could explain why distantly related duplicates often show different mutational effects (Conant and Wagner, 2004).

### Freezing survival is not affected by tps-2 silencing

There was no statistical difference between the proportion of moving nematodes after freezing of non-treated samples compared to that of *Pd-tps-2a(RNAi)-*treated samples (Fig. 2E). The lack of freezing sensitivity in RNAi-treated samples is not surprising given that the trehalose content was not decreased (Fig. 2D). It is also likely that genes other than those involved in trehalose synthesis are involved in freezing survival.

Taken together, the characteristics of gene duplicates explain the different RNAi sensitivity between the two *tps-2* genes in PaDAW1 as well as the lack of effects on the trehalose content. The fact that not only the two *tps-2* genes, but also *gob-1* is involved in trehalose synthesis, indicates a multiple backup system in PaDAW1, underlining the importance of this sugar as a cryoprotectant against environmental stressors common in Antarctica.

## MATERIALS AND METHODS

### Nematode culturing and cold acclimation

PaDAW1 was originally isolated from McMurdo Sound region, Antarctica (Wharton and Brown, 1989) and has been maintained in the laboratory for more than 25 years (Raymond et al., 2013b). Mixed nematode cultures were grown on *Escherichia coli*-seeded NGM agar plates at 20°C and subcultured weekly. Nematodes used for these experiments were collected from culture plates by a modified Baermann technique (Flegg and Hooper, 1970), and subcultured in five replicates of exactly equal volume. They were first incubated at 20°C for 3 days and then at 5°C for another 24 h. After cold acclimation, nematodes were re-collected, snap frozen in a mixture of dry ice and ethanol, and stored at −80°C until analysis.

### RNA isolation and cDNA synthesis

RNA was extracted using TRIzol^®^ Reagent (Ambion, Foster City, CA, USA) and RNeasy^®^ Mini Kits (Qiagen, Hilden, Germany) and reverse-transcribed using the VILO cDNA synthesis kit (Invitrogen, Carlsbad, CA, USA) as described previously (Seybold et al., 2016).

### Candidate gene cloning for RNAi

Candidate genes were selected using two different sets of data: a study of *P. murrayi* (Adhikari et al., 2009) and transcriptomic data from PaDAW1 (Thorne et al., 2014) (Table S2). We selected genes that showed significant upregulation on exposure to freezing in both *P. murrayi* and PaDAW1, particularly those involving trehalose metabolism. Target genes were PCR amplified from cDNA with gene specific primers using Taq DNA Polymerase dNTPack (Roche, Basel, Switzerland) as described previously (Seybold et al., 2016) and Table S1.

Two sets of transcripts homologous to *tps* were identified in the PaDAW1 dataset. Initial analyses suggested these were two non-overlapping portions of the same gene (Thorne et al., 2014). However, a search of the genomic scaffolds indicates that they probably come from different regions of the genome and probably represent a gene duplication. These two regions were termed *tps-2a*, showing a larger alignment, and *tps-2b*, a smaller alignment (Table S1).

### RNAi

Double-stranded RNA for soaking experiments was produced by *in vitro* transcription of PCR products using the MEGAscript^®^T7 Transcription Kit (Life Technologies, Carlsbad, CA, USA). RNA uptake was performed by desiccating nematodes at 98% relative humidity and 20°C for 24 h, prior to soaking in dsRNA solutions as described previously (Seybold et al., 2016). Nematode cultures (four replicates for each treatment) were then rehydrated in soaking buffer (plus 1 mg/ml dsRNA, and minus dsRNA) for 16 h. Cultures were then incubated for 24 h at 5°C for recovery before they were harvested and processed.

### qPCR

Quantitative PCR was performed using the BioRad CFX96 System (Hercules, CA, USA) and BioRad SSoFast EVA Green Supermix with Low Rox. A typical 20 µl reaction contained a 5 µl sample (total of 50 ng cDNA), 10 µl SYBR green mix, 1.2 µl primer mix and 3.2 µl mQ water. Specificity and efficiency assays were performed for all genes (Fig. S2 and Supplementary Methods). Of six housekeeping genes tested, the combination of *Pd-gpd-2* and *Pd-tba-1* was defined as the most stable and used for all qPCR experiments to normalize data from each individual assay. The genes tested were *tps-2*, *gob-1*, *lea-1* (late embryogenesis abundant protein 1), *hsp-70* and *gpx-1*. BioRad FCX Manager software was used to control qPCR settings and to analyze qPCR data as described previously (Seybold et al., 2016). Primer details for qPCR analyses can be found in Table S2 and Seybold et al. (2016).

Relative differences in expression of target genes were assessed using the ΔΔC_t_ (Livak) method (Livak and Schmittgen, 2001), and the relative difference in expression of the target gene in different samples was determined. This involves normalizing the C_t_ of the target gene to that of the reference genes (Table S3), for both the test sample and the control sample (producing a normalized relative expression value ΔC_t_). In the next step, the ΔC_t_ of the test sample is normalized to that of the control sample to give a measure of relative expression. This approach produces an estimate of how test gene expression changed with respect to the control condition and corrects for differences in the RNA yield of each culture that would otherwise complicate the analysis.

### Gas chromatography

Sugar extraction was performed by using a technique modified from Wharton et al. (2000). Sugars were extracted to compare trehalose levels from both cold-acclimated and non-acclimated cultures (grown at 5°C and 20°C) as well as from *Pd-tps-2(RNAi)*-treated and non-treated (control) samples acclimated at 5°C. RNAi cultures were plated out on NGM agar plates and incubated for 72 h, to allow time for changes in trehalose levels. After incubation, samples were collected as described above and snap frozen in a mixture of dry ice and ethanol before processing.

After extraction (Wharton et al., 2000), the sugars in dried samples were converted to their trimethylsilyl derivatives by adding 20 µl Sylon (Supelco, Sigma-Aldrich) and incubation for 5 min at room temperature. Derivatized samples (15 µl) were injected onto the column of an Agilent 6890N Network gas chromatograph. To reduce variation between replicas caused by the injection technique, hot needle injection after Barwick (1999) was performed. Briefly, after insertion into the injection zone, the needle is allowed to heat up for 5 s. The sample is then rapidly injected by pushing down the plunger and the needle quickly withdrawn from the inlet within 1 s. Sugars were identified and quantified using reference standards. Recovery of sugars was estimated by inclusion of 240 µg of Dulcitol in the original nematode sample and analysis of the Dulcitol peak (Wharton et al., 2000) (Supplementary Information).

### Freezing survival

The freezing survival experiment was performed according to Smith et al. (2008). Following RNAi soaking and acclimation at 5°C for 24 h, nematodes were washed off the plates and 50 µl samples of nematode suspension were transferred into 0.5 ml microcentrifuge tubes. These were transferred to a cooling block (Wharton et al., 2004b) and cooled from 1°C to −15°C at 0.5 min^−1^. Freezing was seeded by adding a small ice crystal to each tube when the temperature was −1°C. Samples were held at −15°C for 30 min, then warmed to 1°C at 0.5 min^−1^, and then were transferred to mQ water in watch-glasses and incubated for 24 h at 20°C. Survival of nematodes was assessed by determining the proportion of moving nematodes. To perform statistical analysis, 3×100 nematodes were counted for each of the three replicates of non-treated and *Pd-tps-2(RNAi)*-treated samples. A *t*-test (parametric, two samples, equal variance) was used to assess the statistical significance of sample differences. Control samples consisted of 50 µl nematode suspension in 0.5 ml microcentrifuge tubes, kept at 20°C for the duration of the experiment.

## Supplementary Material

Supplementary information
