# Supplementary material for: Investigating trehalose synthesis genes after cold acclimation in the Antarctic nematode Panagrolaimus sp. DAW1
Source: Biol Open. 2017 Nov 24;6(12):1953–9. doi: 10.1242/bio.023341 (PMC5769639; doi:10.1242/bio.023341)
Supplement: Supplementary information [file biolopen-6-023341-s1.pdf]

PCR primers for amplification of genes for dsRNA production

Primer name	Sequence (5' to 3')	Tm (°C)	Start (bp)	End (bp)	Clone size (bp)	cDNA size (bp)	Pd number
Pd-tps-2a F	TGCTTGAGTTTGTGAATCTGGG	66.2	788	1321	534	1564	PdU054198_v1.1
Pd tps-2a R	ACGCTACAAGTTTATCATCCAG	59.7					
Pd-tps-2b F	AACTCCCAATGAACATGACCA	64.1	849	1369	520	1432	PdU054346_v1.1
Pd-tps-2b R	CGTTGTTGTTGAAGAAGGTGAT	62.8					

Table S1: Genes and PCR primers used to amplify PaDAW1 genes for dsRNA production in *E. coli*. In addition to the gene names and primer sequences, primer melting points, clone location and size, cDNA size, and the PaDAW1 contig name [1] is shown. The cDNA size refers to the size of the gene identified at each Pd number.

qPCR primers

qPCR primers were designed and prepared as described for PCR primers, except the product size range was set to 80-120 bp. Although the maximum temperature difference of primer pairs was set to 1 °C using Primer3web, the final temperature difference of some primer pairs exceeded this value. Sequences for all qPCR primers are shown in Table S2.

Primer name	Sequence (5' to 3')	Tm (°C)	Use	Efficiency (%)
Pd-gob-1 F	CATTCCAACTCCATCAGCTTTATT	64.0	qPCR validation	99.4
Pd-gob-1 R	TGATCCATCGACAGAATTAGAAGT	62.6	qPCR validation	99.4
Pd-gpd-2 F	TGTGTTGCTGTTAATGATCCTT	61.0	Reference gene	98.4
Pd-gpd-2 R	CAAGATTTCCACCTTCTGCTTT	63.2	Reference gene	98.4
Pd-gpx-1 F	TGATCGTGGATTTGAAGTTGC	64.9	qPCR validation	97.8
Pd-gpx-1 R	TATCAATTTCACATGCAGGTTCC	64.6	qPCR validation	97.8
Pd-hsp-70 F	CTGAAACTCTTACTCGTGCCA	62.0	qPCR validation	99.4
Pd-hsp-70 R	CAGCATCTTCCATAACCTTTTGA	63.9	qPCR validation	99.4
Pd-lea-1 F	AAAGAAAGTGCCCAAAATGC	60.7	qPCR validation	108.8
Pd-lea-1 R	CTTTATCAGAAGCATGTTGAACT	62.8	qPCR validation	108.8
Pd-tba-1 F	CCGAGGGGATGTTGTACCTA	63.6	Reference gene	95.6
Pd-tba-1 R	TAAAGCCAGTTGGACACCAAT	63.1	Reference gene	95.6
Pd-tps-2a F	AGTTTCAGAATCACCACAGACG	63.1	qPCR validation	109.3
Pd-tps-2a R	TGATAAAGGGCAGGGCATTG	66.9	qPCR validation	109.3
Pd-tps-2b F	ACCAAACCAGCAGAGGGATT	64.7	qPCR validation	103.7
Pd-tps-2b R	ATCGGAGTTGTCACCCCAAA	66.5	qPCR validation	103.7

Table S2: Names, sequences, use and efficiency of qPCR primers. Primer names are formed according to the following rules: Species name (e.g. *Pd* = PaDAW1, - gene (e.g. *gob*) - primer (F = forward, R = reverse).

Supplementary Methods

Specificity and efficiency assays

Specificity assay: To ensure that primers amplify the expected product size range of 80-120 bp and that there was no amplification from any contaminating genomic DNA, primers were tested using standard PCR. PCR products were analysed via agarose gel electrophoresis for primer specificity (single band of the expected size), for genomic DNA contamination (no band in the -RT control and no or a bigger band in the gDNA control) and general contamination (water only control). Figure S1 presents an example of such an agarose gel.

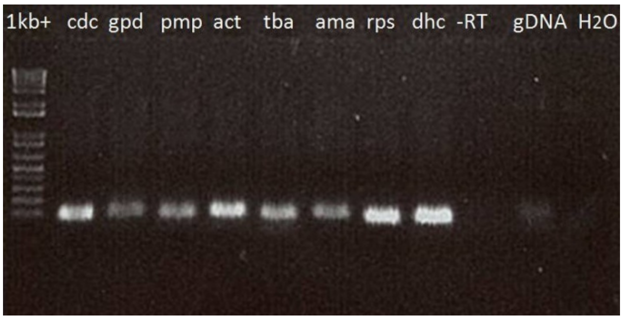

Figure S1: Agarose gel showing single bands of the expected size for PCR products using 8 primer pairs, no band in the -RT control, a very faint band in the gDNA control and no band in the water only control.

Melting curves (step f in the qPCR program) were also performed for each individual gene. They were analysed for primer specificity (indicated by a single melting peak) and are listed below.

Efficiency assay: Standard curves were performed for each individual gene (technical triplicates) using a dilution series of cDNA (1×, 10×, 100×, 1000×, 10000×). For a qPCR assay the efficiency should be between 90% and 110%. This means that for every PCR cycle there is a doubling of the amount of product. Primers with efficiencies outside the acceptable range were discarded. qPCR primers and their efficiencies are listed in Table S1 and efficiency assays are listed below.

In each graph the amplification data is plotted with the log cDNA concentration on the x-axes and the threshold cycle on the y-axes. The threshold cycle is the cycle at which the fluorescence signal is first detected at higher than background levels. The software (BioRad FCX Manager) calculates efficiency (E), fit to the curve (R^2) and slope of the curve (Slope). A R^2 value between 0.95 and 1.0 indicates accurate efficiency measurement and a slope of -3.6 and -3.1 equals an efficiency of 90-100%.

Biology Open • Supplementary information

Pd-gob-1

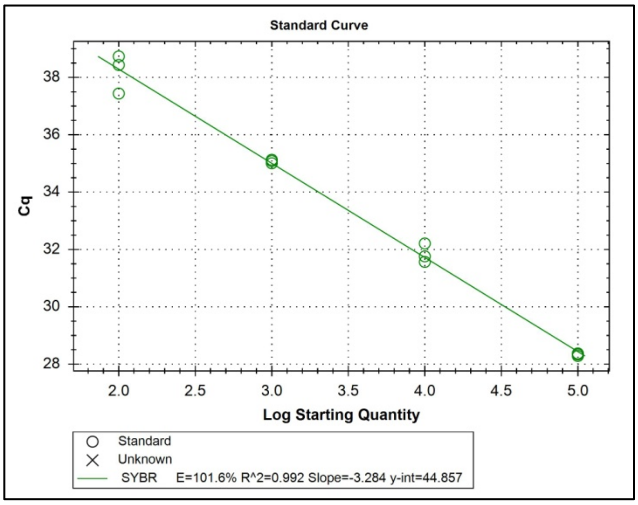

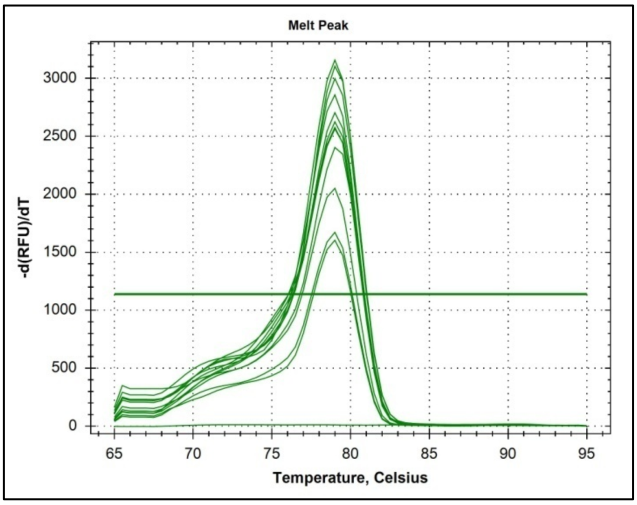

Pd-gpd-2

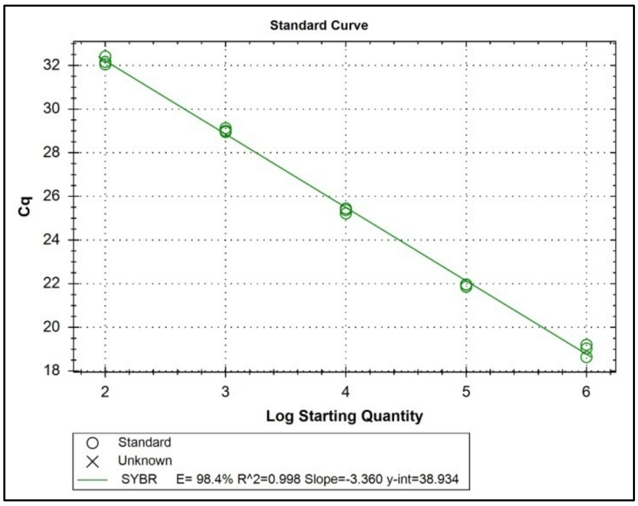

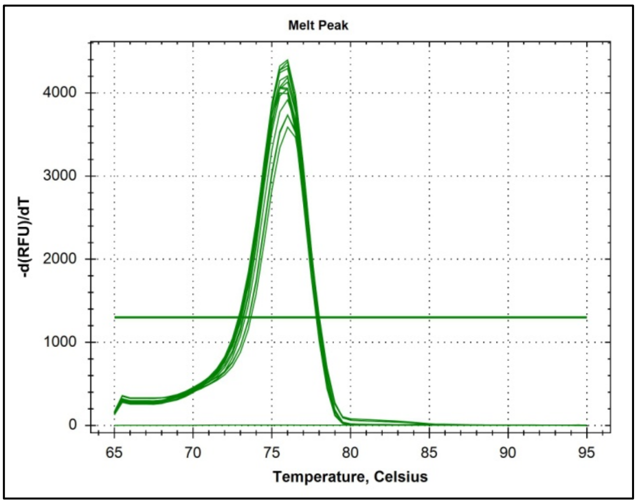

Pd-gpx-1

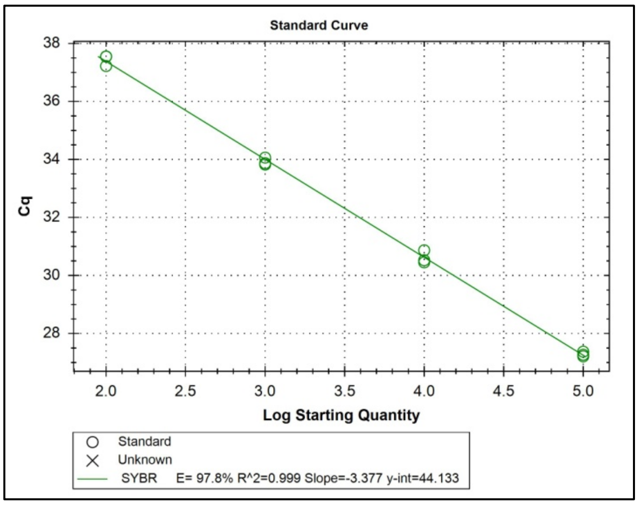

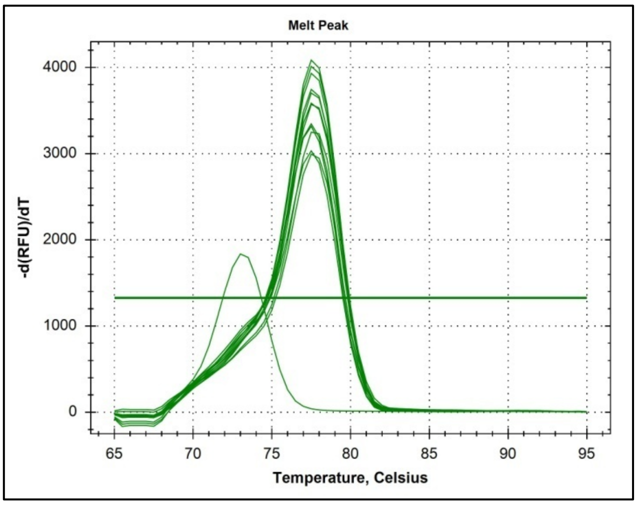

Biology Open • Supplementary information

Pd-hsp-70

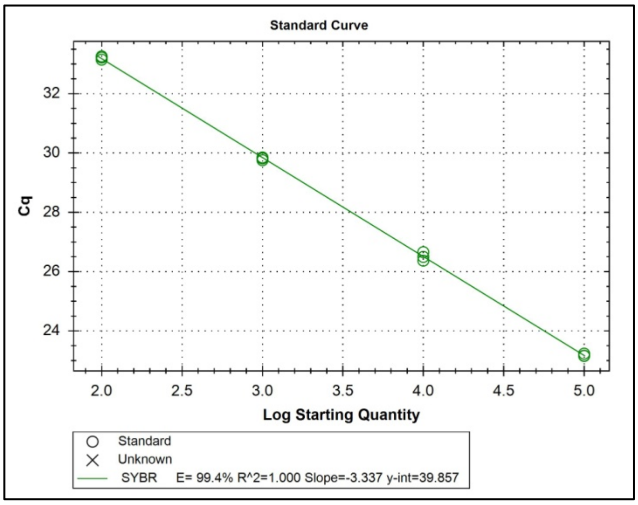

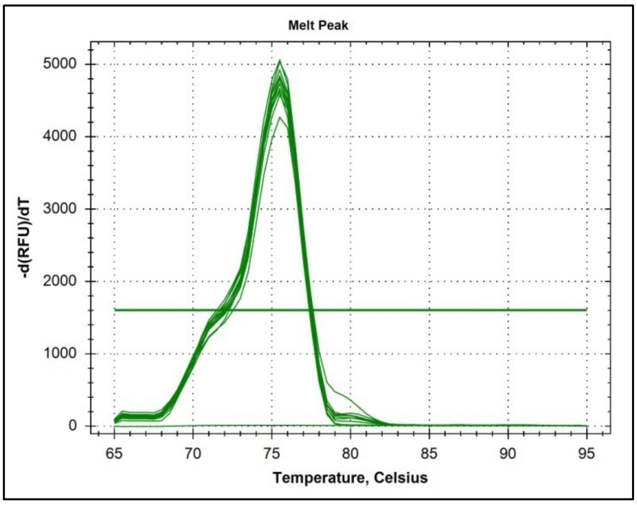

Pd-lea-1

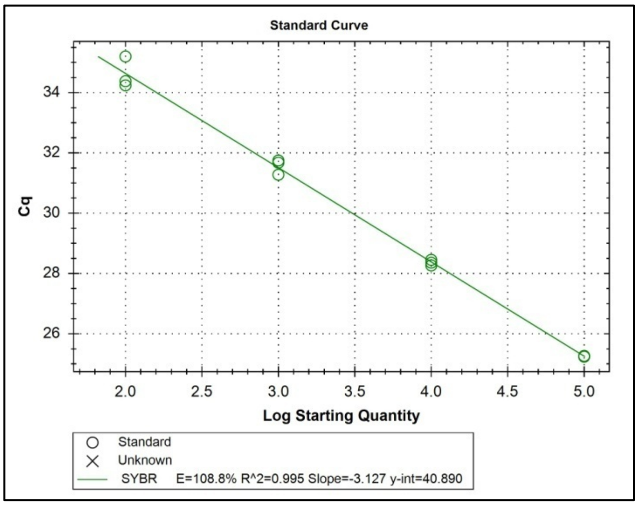

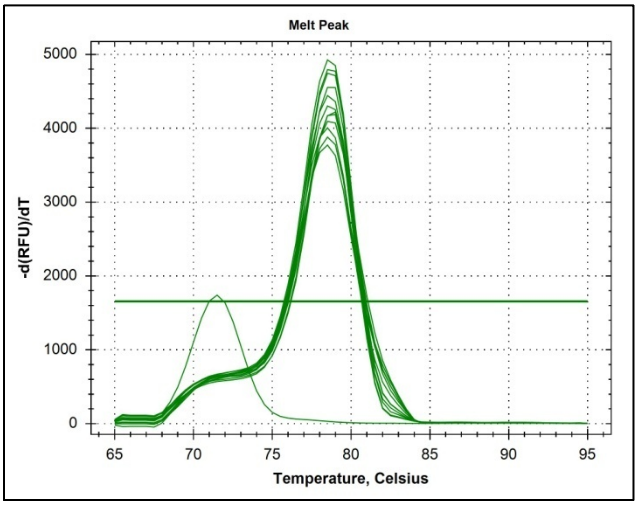

Pd-tba-1

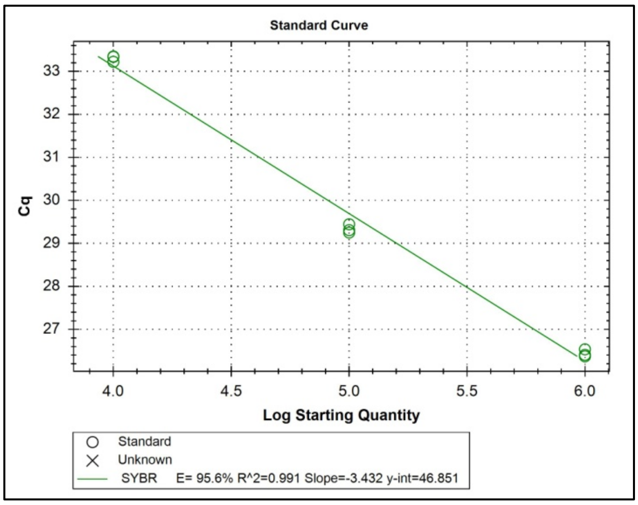

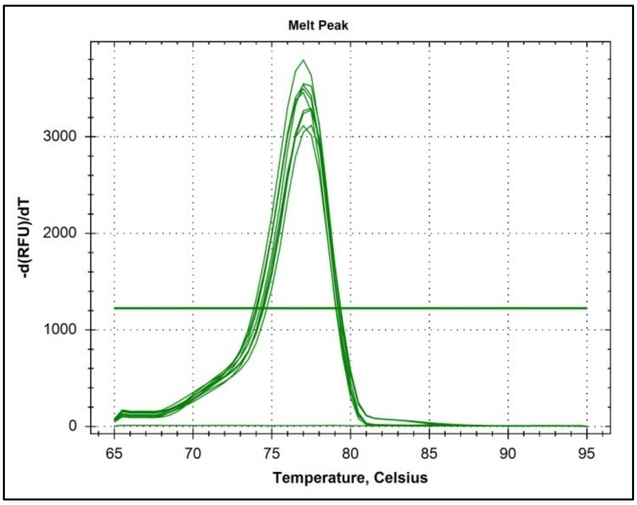

Biology Open • Supplementary information

Pd-tps-2a

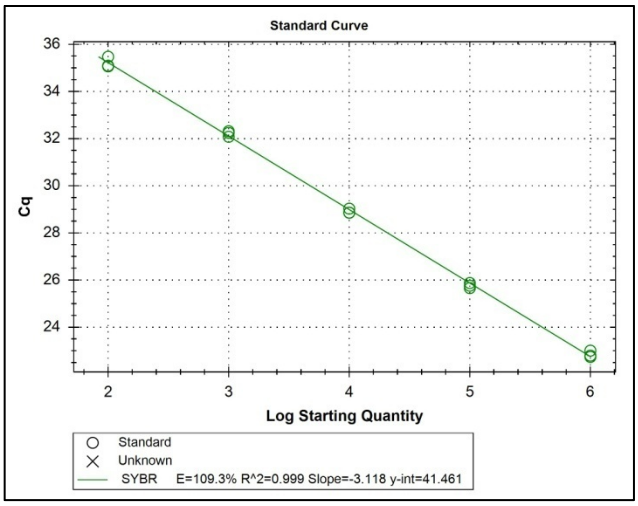

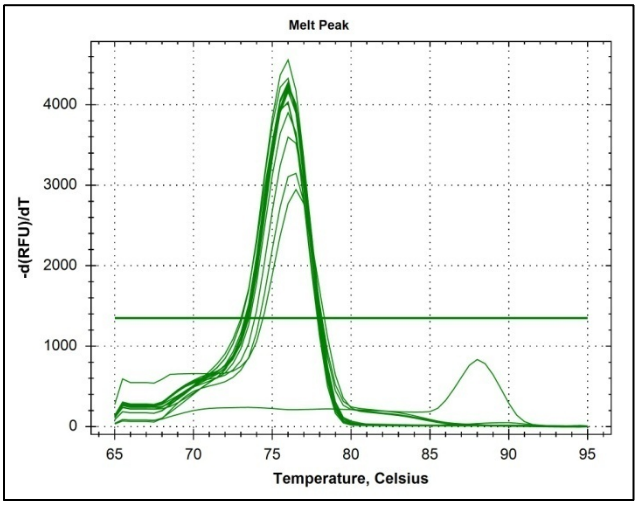

Pd-tps-2b

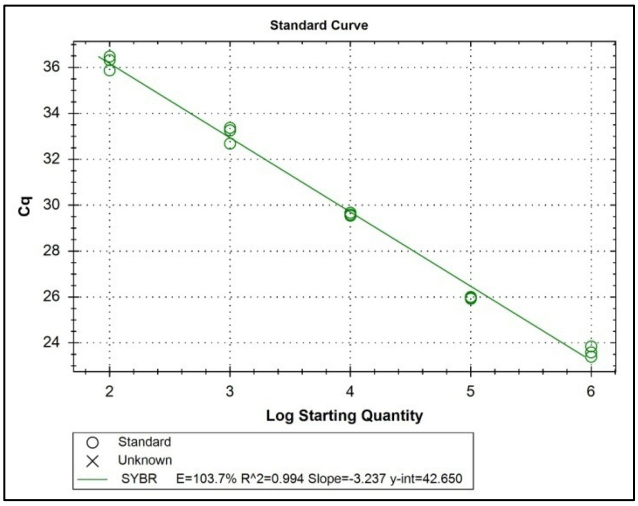

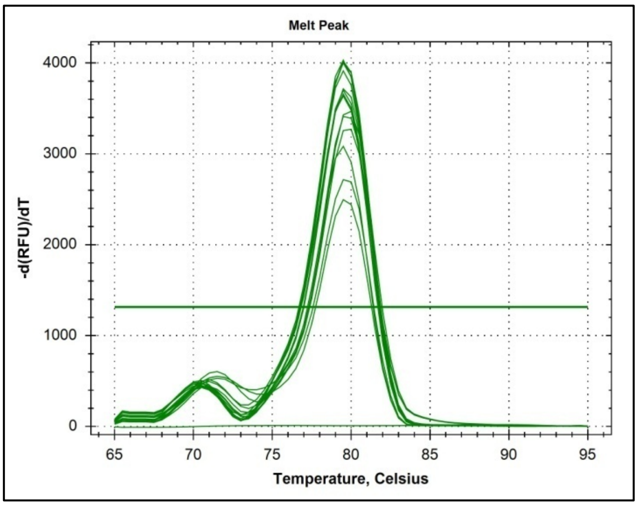

Biology Open • Supplementary information

Reference genes

Reference genes for PaDAW1 were selected by searching the literature for stable reference genes in the closely-related *C. elegans*. Primers were designed for the following six reference genes (as described above): *Pd-act-1, Pd-ama-1, Pd-cdc-42, Pd-gpd-2, Pd-pmp-3* and *Pd-tba-1*. Of this group, the most stable combination of two reference genes were defined by using NormFinder (Table S2).

This algorithm estimates the stability of gene expression among groups, called the stability value. Genes that deviate the least from the calculated values are the most stable. With a stability value of 0.024, the combination of *Pd-gpd-2* and *Pd-tba-1* was defined as the most stable and used for all qPCR experiments. Because the suitability of selected reference genes can differ between experiments, the stability value in each experiment was analysed via NormFinder [2].

Gene name	Stability value		
Pd-cdc-42	0.049	**Best gene**	*Pd-gpd-2*
Pd-gpd-2	0.027	**Stability value**	0.027
Pd-pmp-3	0.057	**Best combination of two genes**	*Pd-gpd-2*
Pd-act-1	0.048		*Pd-tba-1*
Pd-tba-1	0.042	**Stability value for best combination for two genes**	0.024
Pd-ama-1	0.031		

Table S3: Stability value of six chosen reference genes for PaDAW1: *Pd-cdc-42*, *Pd-gpd-2*, *Pd-pmp-3*, *Pd-act-1*, *Pd-tba-1* and *Pd-ama-1*. The most stable gene is *Pd-gpd-2* (stability value 0.027) and the most stable gene combination is *Pd-gpd-2* and *Pd-tba-1* (stability value 0.024) [3,4]

Biology Open • Supplementary information

Example chromatograms

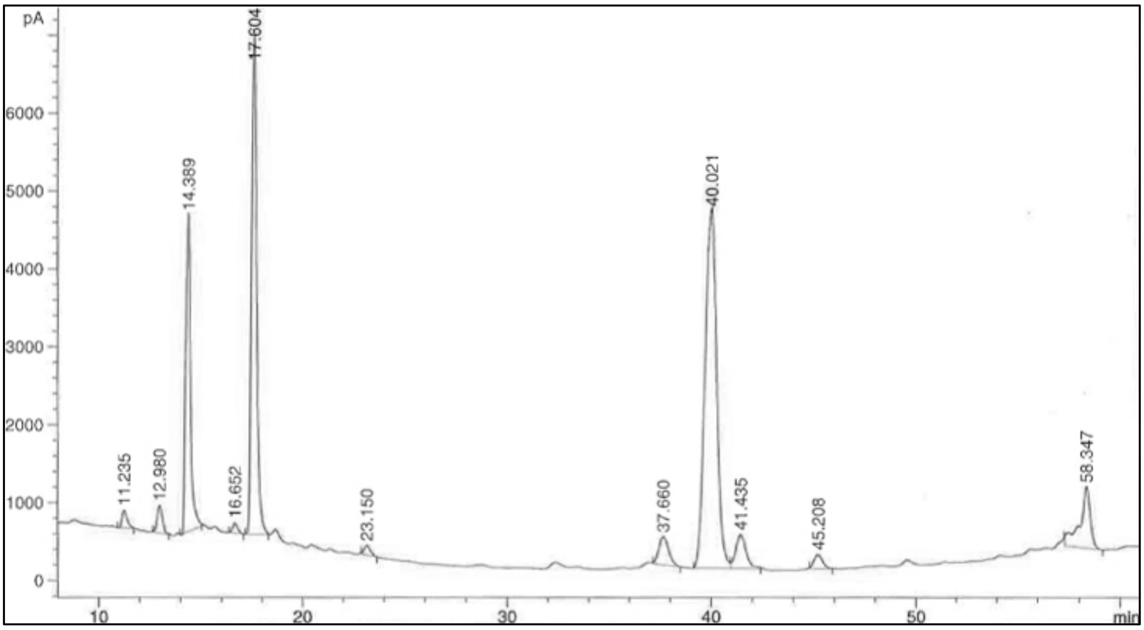

Example chromatogram of a 20°C sample (elution times: 17 = Glycerol, 40 = Dulcitol (internal standard), 58 = Trehalose)

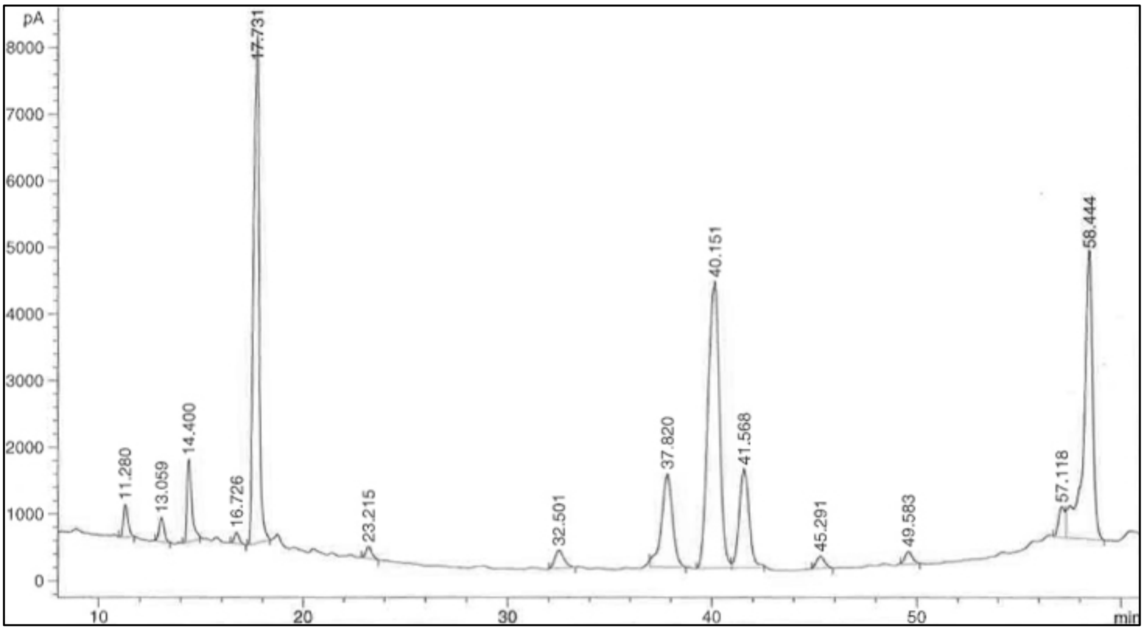

Example chromatogram of a 5°C sample

Biology Open • Supplementary information

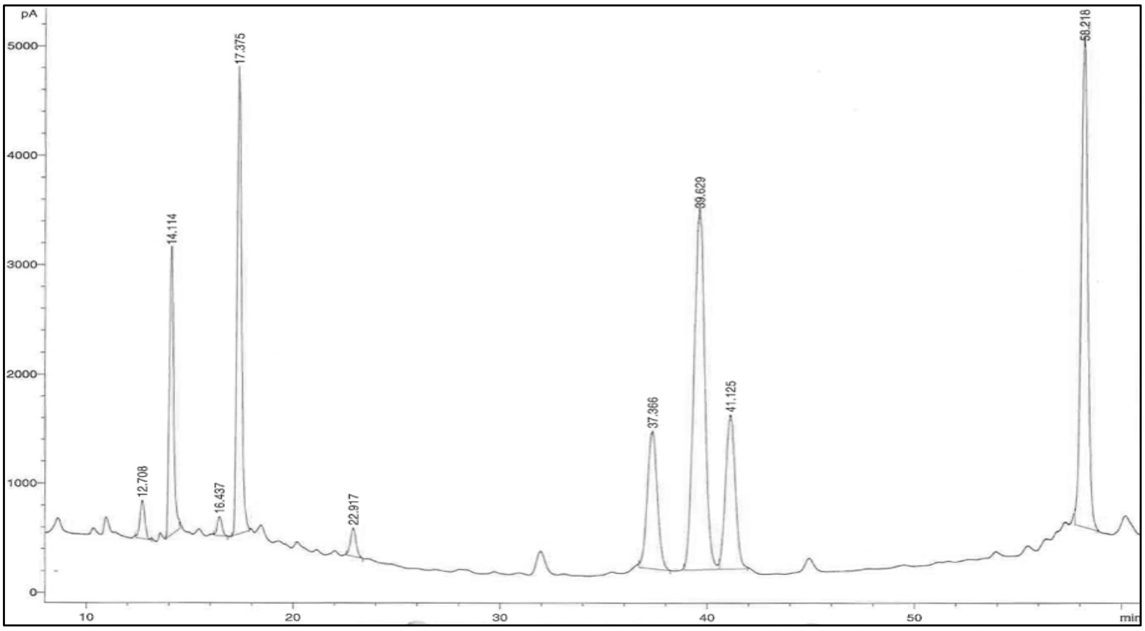

Example chromatogram of a non-treated acclimated (control) sample (elution times: 17 = Glycerol, 39 = Dulcitol (internal standard), 58 = Trehalose)

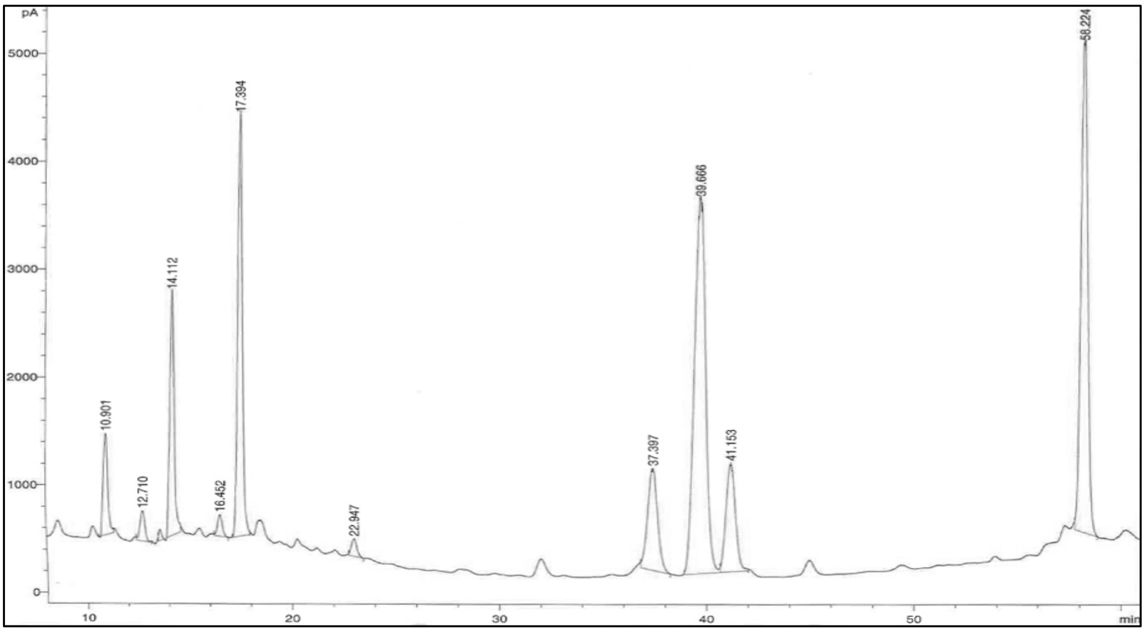

Example chromatogram of a *Pd-tps-2a(RNAi)* treated acclimated sample

References

1. Thorne MAS, Kagoshima H, Clark MS, Marshall CJ, Wharton DA. Molecular analysis of the cold tolerant Antarctic nematode, *Panagrolaimus davidi*. PLoS ONE. 2014;9: e104526–e104526. doi:10.1371/journal.pone.0104526

2. Bustin SA, Benes V, Garson JA, Hellemans J, Huggett J, Kubista M, et al. The MIQE guidelines: minimum information for publication of quantitative real-time PCR experiments. Clin Chem. American Association for Clinical Chemistry; 2009;55: 611–622. doi:10.1373/clinchem.2008.112797

3. Seybold AC, Wharton DA, Thorne MAS, Marshall CJ. Establishing RNAi in a Non-Model Organism: The Antarctic Nematode *Panagrolaimus* sp. DAW1. PLoS ONE; 2016;11: e0166228. doi:10.1371/journal.pone.0166228

4. Seybold A. Molecular Adaptation Mechanisms in the Antarctic Nematode *Panagrolaimus davidi*. Phd Thesis, University of Otago. 2015.

Biology Open • Supplementary information
